# 4-Meth­oxy-*N*-[(4-methyl­phen­yl)sulfon­yl]benzamide including an unknown solvate

**DOI:** 10.1107/S1600536813028158

**Published:** 2013-10-19

**Authors:** Swamy Sreenivasa, Bandrehalli Siddagangaiah Palakshamurthy, Jagdish Tonannavar, Yenagi Jayashree, Achar Gurumurthy Sudha, Parameshwar Adimoole Suchetan

**Affiliations:** a Department of Studies and Research in Chemistry, Tumkur University, Tumkur, Karnataka 572 103, India; b Department of Studies and Research in Physics, U.C.S., Tumkur University, Tumkur, Karnataka 572 103, India; c Department of Physics, Karnatak University, Dharwad, Karnataka 580 003, India; d University College of Science, Tumkur University, Tumkur, India; e Department of Studies and Research in Chemistry, U.C.S., Tumkur University, Tumkur, Karnataka 572 103, India

## Abstract

In the title compound, C_15_H_15_NO_4_S, the dihedral angle between the benzene rings is 78.62 (16)°. In the crystal, adjacent mol­ecules are linked along the *c* axis into *C*(4) chains through strong N—H⋯O hydrogen bonds. Mol­ecules are further connected through C—H⋯O hydrogen bonds into a hexa­meric unit generating an *R* ^6^_6_(66) motif. Another C—H⋯O inter­action connects the mol­ecules along the *c* axis, forming *C*(5) chains. A region of disordered electron density, most probably disordered methanol–water solvent mol­ecules, was treated with the SQUEEZE routine in *PLATON* [Spek (2009 ▶). *Acta Cryst*. D**65**, 148–155]. The formula mass and unit-cell characteristics do not take into account this disordered solvent.

## Related literature

For similar structures, see: Gowda *et al.* (2009 ▶); Suchetan *et al.* (2010*a* ▶,*b* ▶,*c* ▶, 2011 ▶); Sreenivasa *et al.* (2013 ▶). For details of the use of the SQUEEZE routine in *PLATON, see:* Spek (2009 ▶).
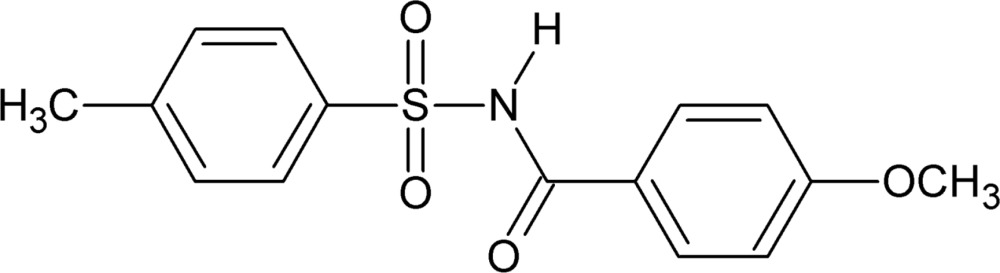


## Experimental

### 

#### Crystal data

C_15_H_15_NO_4_S
*M* *_r_* = 305.34
Trigonal, 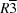

*a* = 27.1686 (16) Å
*c* = 10.8594 (6) Å
*V* = 6941.8 (7) Å^3^
*Z* = 18
Mo *K*α radiation
μ = 0.22 mm^−1^
*T* = 293 K
0.32 × 0.27 × 0.19 mm

#### Data collection

Bruker APEXII diffractometer
7701 measured reflections
2106 independent reflections
1604 reflections with *I* > 2σ(*I*)
*R*_int_ = 0.031
θ_max_ = 22.9°

#### Refinement

*R*[*F* ^2^ > 2σ(*F* ^2^)] = 0.039
*wR*(*F* ^2^) = 0.109
*S* = 1.06
2106 reflections
196 parameters
1 restraint
H atoms treated by a mixture of independent and constrained refinement
Δρ_max_ = 0.17 e Å^−3^
Δρ_min_ = −0.31 e Å^−3^

### 

Data collection: *APEX2* (Bruker, 2009 ▶); cell refinement: *APEX2* and *SAINT-Plus* (Bruker, 2009 ▶); data reduction: *SAINT-Plus* and *XPREP* (Bruker, 2009 ▶); program(s) used to solve structure: *SHELXS97* (Sheldrick, 2008 ▶); program(s) used to refine structure: *SHELXL97* (Sheldrick, 2008 ▶); molecular graphics: *Mercury* (Macrae *et al.*, 2008 ▶); software used to prepare material for publication: *SHELXL97*.

## Supplementary Material

Crystal structure: contains datablock(s) I, New_Global_Publ_Block. DOI: 10.1107/S1600536813028158/su2649sup1.cif

Structure factors: contains datablock(s) I. DOI: 10.1107/S1600536813028158/su2649Isup2.hkl

Supplementary material file. DOI: 10.1107/S1600536813028158/su2649Isup3.cml

Additional supplementary materials:  crystallographic information; 3D view; checkCIF report

## Figures and Tables

**Table 1:** Hydrogen-bond geometry (Å, °)

*D*—H⋯*A*	*D*—H	H⋯*A*	*D*⋯*A*	*D*—H⋯*A*
N1—H1*N*⋯O3^i^	0.82 (2)	2.26 (2)	3.038 (3)	160 (3)
N1—H1*N*⋯O2^i^	0.82 (2)	2.59 (3)	3.140 (3)	126 (2)
C10—H10⋯O3^i^	0.93	2.58	3.249 (3)	129
C15—H15*A*⋯O1^ii^	0.96	2.56	3.454 (4)	154

Symmetry codes: (i) 
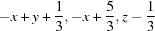
; (ii) 
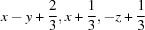
.

**Fig. 1.**
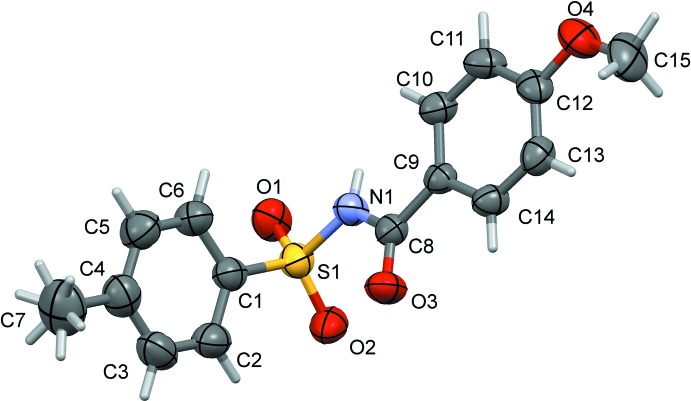
The molecular structure of the title molecule, with atom labelling. Displacement ellipsoids are drawn at the 50% probability level.

**Fig. 2.**
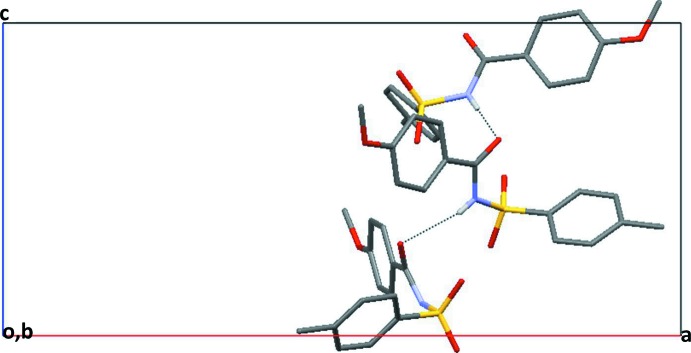
A view along the b axis of the crystal packing of the title compound, with hydrogen bonds shown as dashed lines (see Table 1 for details; C bound hydrogen atoms have been omitted for clarity).

**Fig. 3.**
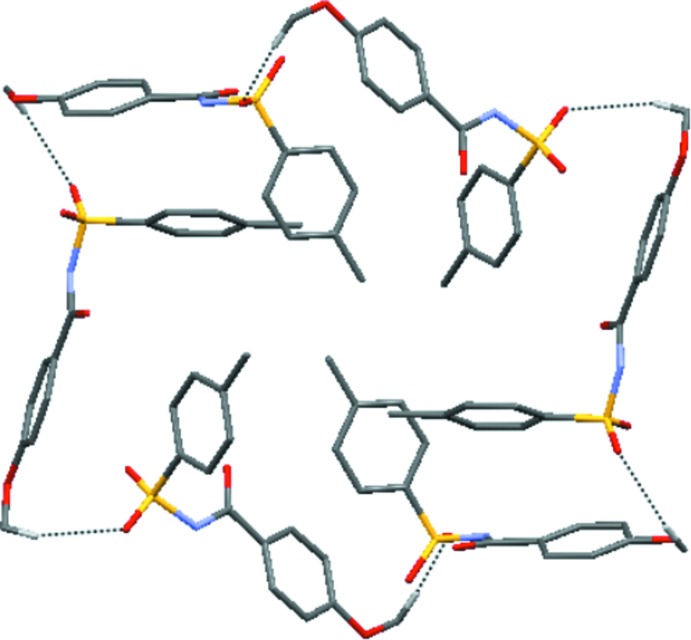
A view of the hexameric C—H···O hydrogen bonded R66(66) ring motif in the crystal structure of the title compound (see Table 1 for details; H atoms not involved in these hydrogen bonds have been omitted for clarity).

**Fig. 4.**
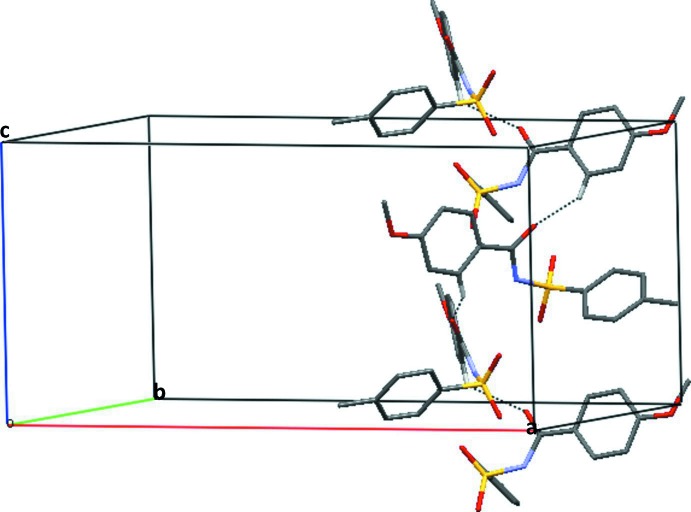
A view of the C—H···O hydrogen bonds linking molecules to form C(5) chains in the crystal structure of the title compound (see Table 1 for details; H atoms not involved in these hydrogen bonds have been omitted for clarity).

## supplementary crystallographic information

### 1. Comment

As a part of our continued efforts to study the crystal structures of *N*-(aroyl)-arylsulfonamides (Sreenivasa *et al.*, 2013 and), we report herein on the crystal structure of the title compound.

In the title compound, Fig. 1, the dihedral angle between the benzene rings is 78.62 (16) °. This is similar to the value of 80.3 (1) ° in *N*-benzoylbenzenesulfonamide (Gowda *et al.*, 2009), 79.4 (1) ° in *N*-benzoyl-4-methylbenzenesulfonamide (Suchetan *et al.*, 2010*a*), and 81.0 (1) ° and 76.3 (1) ° in the two independent molecules of *N*-(4-Chloro-benzoyl)-4-methylbenzenesulfonamide (Suchetan *et al.*, 2010*b*). Interestingly, in 4-methyl-*N*-(4-methylbenzoyl)benzenesulfonamide (Suchetan *et al.*, 2010*c*) the angle is much larger, 89.0 (1) °, similar to the value of 89.8 (1) ° in 4-Methyl-*N*-(4-nitrobenzoyl)benzenesulfonamide (Suchetan *et al.*, 2011), and 88.9 (1) ° in *N*-(3-Methoxybenzoyl)-4-methylbenzenesulfonamide (Sreenivasa *et al.*, 2013).

In the crystal, adjacent molecules are linked along the *c* axis into C(4) chains *via* N—H···O hydrogen bonds (Table 1 and Fig. 2). Molecules are also connected through C—H···O hydrogen bonds into a hexameric unit generating an *R*^6^_6_(66) motif (Table 1 and Fig. 3). Further C—H···O hydrogen bonds connect the molecules along the *c* axis forming C(5) chains (Table 1 and Fig. 4).

### 2. Experimental

The title compound was prepared by refluxing a mixture of 4-methoxybenzoic acid, 4-methylbenzenesulfonamide and phosphorous oxychloride (POCl_3_) for 2 h on a water bath. The resultant mixture was cooled and poured into ice cold water. The solid obtained was filtered and washed thoroughly with water and then dissolved in sodium bicarbonate solution. The compound was then re-precipitated by acidifying the filtered solution with dilute HCl. The compound obtained was filtered and then dried (*M*.p. = 393 K). Colourless prism-like crystals of the title compound were obtained by slow evaporation of an water/methanol solution (1:1) at room temperature.

### 3. Refinement

The NH hydrogen atom was located in a difference Fourier map and refined with a distance restraint of N—H = 0.86 (2) Å. The C bound H atoms were positioned with idealized geometry and refined using a riding model: C—H = 0.93–0.96 Å with *U*_eq_ = 1.5*U*_eq_(*C*-methyl) and = 1.2*U*_eq_(C) for other H atoms. The C7 methyl H atoms were refined with AFIX 127, *viz*., an idealized disordered methyl group with two positions rotated from each other by 60 °. The crystal did not diffract significantly beyond 22 ° in θ. A region of disordered electron density, probably disordered methanol/water solvent molecules, was treated with the SQUEEZE routine in *PLATON* (Spek, 2009); more details are given in "_platon_squeeze_details".

### Crystal data

**Table:**

C_15_H_15_NO_4_S	*D*_x_ = 1.315 Mg m^−^^3^
*M**_r_* = 305.34	Mo *K*α radiation, λ = 0.71073 Å
Trigonal, *R*3	Cell parameters from 1123 reflections
Hall symbol: -R 3	θ = 0.0–22.9°
*a* = 27.1686 (16) Å	µ = 0.22 mm^−^^1^
*c* = 10.8594 (6) Å	*T* = 293 K
*V* = 6941.8 (7) Å^3^	Prism, colourless
*Z* = 18	0.32 × 0.27 × 0.19 mm
*F*(000) = 2880	

### Data collection

**Table:**

Bruker APEXII diffractometer	1604 reflections with *I* > 2σ(*I*)
Radiation source: fine-focus sealed tube	*R*_int_ = 0.031
Graphite monochromator	θ_max_ = 22.9°, θ_min_ = 1.5°
phi and ω scans	*h* = −28→27
7701 measured reflections	*k* = −29→27
2106 independent reflections	*l* = −11→11

### Refinement

**Table:**

Refinement on *F*^2^	Primary atom site location: structure-invariant direct methods
Least-squares matrix: full	Secondary atom site location: difference Fourier map
*R*[*F*^2^ > 2σ(*F*^2^)] = 0.039	Hydrogen site location: inferred from neighbouring sites
*wR*(*F*^2^) = 0.109	H atoms treated by a mixture of independent and constrained refinement
*S* = 1.06	*w* = 1/[σ^2^(*F*_o_^2^) + (0.050*P*)^2^ + 5.9239*P*] where *P* = (*F*_o_^2^ + 2*F*_c_^2^)/3
2106 reflections	(Δ/σ)_max_ = 0.001
196 parameters	Δρ_max_ = 0.17 e Å^−^^3^
1 restraint	Δρ_min_ = −0.31 e Å^−^^3^

### Special details

**Table:**

Geometry. All e.s.d.'s (except the e.s.d. in the dihedral angle between two l.s. planes) are estimated using the full covariance matrix. The cell e.s.d.'s are taken into account individually in the estimation of e.s.d.'s in distances, angles and torsion angles; correlations between e.s.d.'s in cell parameters are only used when they are defined by crystal symmetry. An approximate (isotropic) treatment of cell e.s.d.'s is used for estimating e.s.d.'s involving l.s. planes.
Refinement. Refinement of *F*^2^ against ALL reflections. The weighted *R*-factor *wR* and goodness of fit *S* are based on *F*^2^, conventional *R*-factors *R* are based on *F*, with *F* set to zero for negative *F*^2^. The threshold expression of *F*^2^ > σ(*F*^2^) is used only for calculating *R*-factors(gt) *etc*. and is not relevant to the choice of reflections for refinement. *R*-factors based on *F*^2^ are statistically about twice as large as those based on *F*, and *R*- factors based on ALL data will be even larger.

### Fractional atomic coordinates and isotropic or equivalent isotropic displacement parameters (Å^2^)

**Table:**

	*x*	*y*	*z*	*U*_iso_*/*U*_eq_	Occ. (<1)
S1	0.64344 (3)	0.92841 (3)	0.07169 (6)	0.0512 (3)	
O2	0.67632 (8)	0.92727 (8)	0.17255 (17)	0.0630 (6)	
O3	0.58608 (8)	0.93575 (8)	0.29224 (17)	0.0589 (6)	
C9	0.57569 (10)	1.01361 (10)	0.2290 (2)	0.0408 (6)	
N1	0.61811 (10)	0.97065 (10)	0.1035 (2)	0.0485 (6)	
H1N	0.6281 (12)	0.9968 (9)	0.055 (2)	0.062 (10)*	
O1	0.66938 (8)	0.94701 (8)	−0.04522 (17)	0.0632 (6)	
O4	0.52572 (9)	1.13258 (9)	0.2828 (2)	0.0741 (7)	
C8	0.59338 (10)	0.97057 (11)	0.2137 (2)	0.0432 (7)	
C10	0.57316 (11)	1.04599 (11)	0.1330 (2)	0.0492 (7)	
H10	0.5831	1.0413	0.0538	0.059*	
C12	0.54143 (11)	1.09243 (11)	0.2710 (3)	0.0504 (7)	
C14	0.56081 (11)	1.02166 (11)	0.3456 (2)	0.0506 (7)	
H14	0.5625	1.0004	0.4107	0.061*	
C11	0.55601 (12)	1.08497 (12)	0.1542 (3)	0.0555 (8)	
H11	0.5543	1.1064	0.0893	0.067*	
C13	0.54340 (11)	1.06075 (12)	0.3676 (3)	0.0543 (8)	
H13	0.5332	1.0655	0.4465	0.065*	
C1	0.58283 (12)	0.86088 (12)	0.0570 (2)	0.0506 (7)	
C3	0.53039 (16)	0.76272 (14)	0.1127 (3)	0.0733 (9)	
H3	0.5270	0.7326	0.1598	0.088*	
C6	0.54185 (13)	0.85199 (13)	−0.0290 (3)	0.0591 (8)	
H6	0.5458	0.8817	−0.0784	0.071*	
C2	0.57735 (14)	0.81618 (14)	0.1276 (3)	0.0640 (9)	
H2	0.6052	0.8220	0.1850	0.077*	
C5	0.49512 (14)	0.79869 (14)	−0.0406 (3)	0.0689 (9)	
H5	0.4670	0.7929	−0.0972	0.083*	
C4	0.48878 (14)	0.75366 (14)	0.0291 (3)	0.0678 (9)	
C15	0.50425 (16)	1.13869 (16)	0.3982 (3)	0.0871 (11)	
H15A	0.4715	1.1032	0.4206	0.131*	
H15B	0.4938	1.1675	0.3915	0.131*	
H15C	0.5330	1.1495	0.4602	0.131*	
C7	0.43705 (17)	0.69489 (15)	0.0145 (4)	0.1057 (14)	
H7A	0.4150	0.6948	−0.0546	0.159*	0.50
H7B	0.4143	0.6851	0.0878	0.159*	0.50
H7C	0.4491	0.6676	0.0011	0.159*	0.50
H7D	0.4373	0.6702	0.0775	0.159*	0.50
H7E	0.4380	0.6799	−0.0649	0.159*	0.50
H7F	0.4031	0.6974	0.0218	0.159*	0.50

### Atomic displacement parameters (Å^2^)

**Table:**

	*U*^11^	*U*^22^	*U*^33^	*U*^12^	*U*^13^	*U*^23^
S1	0.0582 (5)	0.0608 (5)	0.0468 (5)	0.0389 (4)	0.0014 (4)	−0.0045 (3)
O2	0.0687 (13)	0.0786 (14)	0.0612 (13)	0.0515 (12)	−0.0164 (10)	−0.0147 (11)
O3	0.0789 (14)	0.0686 (13)	0.0476 (12)	0.0506 (11)	0.0087 (10)	0.0122 (10)
C9	0.0383 (15)	0.0458 (15)	0.0404 (15)	0.0226 (13)	−0.0015 (12)	−0.0007 (12)
N1	0.0642 (16)	0.0548 (15)	0.0397 (14)	0.0396 (13)	0.0062 (12)	0.0032 (12)
O1	0.0702 (13)	0.0730 (13)	0.0552 (13)	0.0423 (11)	0.0176 (11)	0.0014 (10)
O4	0.0859 (15)	0.0791 (15)	0.0861 (17)	0.0629 (14)	−0.0009 (12)	−0.0053 (12)
C8	0.0431 (16)	0.0488 (17)	0.0395 (16)	0.0243 (14)	−0.0013 (13)	−0.0007 (13)
C10	0.0507 (17)	0.0638 (19)	0.0421 (16)	0.0353 (15)	0.0030 (13)	0.0049 (14)
C12	0.0429 (16)	0.0537 (18)	0.063 (2)	0.0305 (14)	−0.0057 (14)	−0.0041 (15)
C14	0.0595 (18)	0.0636 (18)	0.0401 (16)	0.0394 (16)	0.0035 (14)	0.0045 (14)
C11	0.0605 (18)	0.068 (2)	0.0528 (19)	0.0431 (17)	0.0042 (14)	0.0140 (15)
C13	0.0551 (18)	0.070 (2)	0.0477 (17)	0.0385 (16)	0.0015 (14)	−0.0099 (15)
C1	0.0648 (19)	0.0591 (18)	0.0426 (16)	0.0421 (16)	0.0018 (14)	−0.0019 (14)
C3	0.100 (3)	0.059 (2)	0.067 (2)	0.045 (2)	0.007 (2)	0.0091 (17)
C6	0.069 (2)	0.062 (2)	0.0522 (19)	0.0367 (18)	−0.0046 (16)	0.0035 (15)
C2	0.085 (2)	0.073 (2)	0.0542 (19)	0.054 (2)	−0.0054 (17)	−0.0012 (17)
C5	0.067 (2)	0.074 (2)	0.064 (2)	0.0334 (19)	−0.0101 (17)	−0.0020 (18)
C4	0.075 (2)	0.061 (2)	0.063 (2)	0.0308 (18)	0.0086 (18)	0.0005 (17)
C15	0.099 (3)	0.102 (3)	0.094 (3)	0.076 (2)	−0.005 (2)	−0.030 (2)
C7	0.102 (3)	0.075 (3)	0.107 (3)	0.019 (2)	0.015 (2)	0.003 (2)

### Geometric parameters (Å, º)

**Table:**

S1—O1	1.4169 (19)	C1—C6	1.379 (4)
S1—O2	1.4235 (19)	C1—C2	1.380 (4)
S1—N1	1.643 (2)	C3—C4	1.373 (4)
S1—C1	1.756 (3)	C3—C2	1.382 (4)
O3—C8	1.214 (3)	C3—H3	0.9300
C9—C14	1.380 (3)	C6—C5	1.373 (4)
C9—C10	1.388 (3)	C6—H6	0.9300
C9—C8	1.479 (4)	C2—H2	0.9300
N1—C8	1.372 (3)	C5—C4	1.374 (4)
N1—H1N	0.817 (17)	C5—H5	0.9300
O4—C12	1.362 (3)	C4—C7	1.519 (5)
O4—C15	1.427 (4)	C15—H15A	0.9600
C10—C11	1.373 (4)	C15—H15B	0.9600
C10—H10	0.9300	C15—H15C	0.9600
C12—C11	1.374 (4)	C7—H7A	0.9600
C12—C13	1.375 (4)	C7—H7B	0.9600
C14—C13	1.382 (4)	C7—H7C	0.9600
C14—H14	0.9300	C7—H7D	0.9600
C11—H11	0.9300	C7—H7E	0.9600
C13—H13	0.9300	C7—H7F	0.9600
			
O1—S1—O2	119.45 (13)	C5—C6—H6	120.4
O1—S1—N1	104.29 (12)	C1—C6—H6	120.4
O2—S1—N1	109.86 (11)	C1—C2—C3	119.8 (3)
O1—S1—C1	109.32 (12)	C1—C2—H2	120.1
O2—S1—C1	108.53 (13)	C3—C2—H2	120.1
N1—S1—C1	104.33 (13)	C6—C5—C4	121.7 (3)
C14—C9—C10	118.5 (2)	C6—C5—H5	119.1
C14—C9—C8	117.7 (2)	C4—C5—H5	119.1
C10—C9—C8	123.8 (2)	C3—C4—C5	118.6 (3)
C8—N1—S1	123.9 (2)	C3—C4—C7	120.2 (3)
C8—N1—H1N	121 (2)	C5—C4—C7	121.1 (3)
S1—N1—H1N	113 (2)	O4—C15—H15A	109.5
C12—O4—C15	119.1 (2)	O4—C15—H15B	109.5
O3—C8—N1	120.2 (2)	H15A—C15—H15B	109.5
O3—C8—C9	123.1 (2)	O4—C15—H15C	109.5
N1—C8—C9	116.7 (2)	H15A—C15—H15C	109.5
C11—C10—C9	120.5 (3)	H15B—C15—H15C	109.5
C11—C10—H10	119.8	C4—C7—H7A	109.5
C9—C10—H10	119.8	C4—C7—H7B	109.5
O4—C12—C11	115.7 (3)	H7A—C7—H7B	109.5
O4—C12—C13	123.8 (3)	C4—C7—H7C	109.5
C11—C12—C13	120.5 (2)	H7A—C7—H7C	109.5
C9—C14—C13	121.4 (3)	H7B—C7—H7C	109.5
C9—C14—H14	119.3	C4—C7—H7D	109.5
C13—C14—H14	119.3	H7A—C7—H7D	141.1
C10—C11—C12	120.2 (3)	H7B—C7—H7D	56.3
C10—C11—H11	119.9	H7C—C7—H7D	56.3
C12—C11—H11	119.9	C4—C7—H7E	109.5
C12—C13—C14	119.0 (3)	H7A—C7—H7E	56.3
C12—C13—H13	120.5	H7B—C7—H7E	141.1
C14—C13—H13	120.5	H7C—C7—H7E	56.3
C6—C1—C2	120.0 (3)	H7D—C7—H7E	109.5
C6—C1—S1	119.9 (2)	C4—C7—H7F	109.5
C2—C1—S1	120.1 (2)	H7A—C7—H7F	56.3
C4—C3—C2	120.7 (3)	H7B—C7—H7F	56.3
C4—C3—H3	119.7	H7C—C7—H7F	141.1
C2—C3—H3	119.7	H7D—C7—H7F	109.5
C5—C6—C1	119.1 (3)	H7E—C7—H7F	109.5
			
O1—S1—N1—C8	−174.5 (2)	C11—C12—C13—C14	0.6 (4)
O2—S1—N1—C8	−45.3 (3)	C9—C14—C13—C12	−0.5 (4)
C1—S1—N1—C8	70.9 (2)	O1—S1—C1—C6	−52.8 (2)
S1—N1—C8—O3	−4.4 (4)	O2—S1—C1—C6	175.4 (2)
S1—N1—C8—C9	176.81 (18)	N1—S1—C1—C6	58.3 (2)
C14—C9—C8—O3	13.2 (4)	O1—S1—C1—C2	124.4 (2)
C10—C9—C8—O3	−166.6 (3)	O2—S1—C1—C2	−7.4 (3)
C14—C9—C8—N1	−168.0 (2)	N1—S1—C1—C2	−124.5 (2)
C10—C9—C8—N1	12.1 (4)	C2—C1—C6—C5	1.8 (4)
C14—C9—C10—C11	−0.2 (4)	S1—C1—C6—C5	179.0 (2)
C8—C9—C10—C11	179.7 (2)	C6—C1—C2—C3	−0.7 (4)
C15—O4—C12—C11	173.4 (3)	S1—C1—C2—C3	−177.9 (2)
C15—O4—C12—C13	−6.9 (4)	C4—C3—C2—C1	−0.6 (5)
C10—C9—C14—C13	0.3 (4)	C1—C6—C5—C4	−1.6 (5)
C8—C9—C14—C13	−179.5 (2)	C2—C3—C4—C5	0.8 (5)
C9—C10—C11—C12	0.3 (4)	C2—C3—C4—C7	−179.3 (3)
O4—C12—C11—C10	179.2 (2)	C6—C5—C4—C3	0.3 (5)
C13—C12—C11—C10	−0.5 (4)	C6—C5—C4—C7	−179.6 (3)
O4—C12—C13—C14	−179.0 (2)		

### Hydrogen-bond geometry (Å, º)

**Table:**

*D*—H···*A*	*D*—H	H···*A*	*D*···*A*	*D*—H···*A*
N1—H1*N*···O3^i^	0.82 (2)	2.26 (2)	3.038 (3)	160 (3)
N1—H1*N*···O2^i^	0.82 (2)	2.59 (3)	3.140 (3)	126 (2)
C10—H10···O3^i^	0.93	2.58	3.249 (3)	129
C15—H15*A*···O1^ii^	0.96	2.56	3.454 (4)	154

Symmetry codes: (i) −*x*+*y*+1/3, −*x*+5/3, *z*−1/3; (ii) *x*−*y*+2/3, *x*+1/3, −*z*+1/3.
